# Psychiatric Aspects of Obesity: A Narrative Review of Pathophysiology and Psychopathology

**DOI:** 10.3390/jcm9082344

**Published:** 2020-07-23

**Authors:** Francesco Weiss, Margherita Barbuti, Giulia Carignani, Alba Calderone, Ferruccio Santini, Icro Maremmani, Giulio Perugi

**Affiliations:** 1 2nd Psychiatric Unit, Department of Clinical and Experimental Medicine, Santa Chiara University Hospital, University of Pisa, 56100 Pisa, Italy; francesco_weiss@libero.it (F.W.); margherita.barbuti@hotmail.com (M.B.); morgana.carignani@gmail.com (G.C.); giulio.perugi@med.unipi.it (G.P.); 2 Endocrinological Unit, Department of Clinical and Experimental Medicine, Obesity and Lipodystrophy Research Center, Santa Chiara University Hospital, University of Pisa, 56100 Pisa, Italy; albacalderone@libero.it (A.C.); ferruccio.santini@med.unipi.it (F.S.)

**Keywords:** psychiatric obesity, emotional dysregulation, executive dysfunction, bipolarity, affective temperaments, food addiction

## Abstract

In the last decades, obesity has become a major concern for clinical and public health. Despite the variety of available treatments, the outcomes remain—by and large—still unsatisfactory, owing to high rates of nonresponse and relapse. Interestingly, obesity is being associated with a growing surge of neuropsychiatric problems, certainly related to the pathogenesis of this condition, and likely to be of great consequence as for its treatment and prognosis. In a neurobiologic direction, a sturdy body of evidence has recently shown that the immune–metabolic–endocrine dyscrasias, notoriously attached to excess body weight/adiposity, affect and impair the morpho-functional integrity of the brain, thus possibly contributing to neuroprogressive/degenerative processes and behavioral deviances. Likewise, in a neuropsychiatric perspective, obesity displays complex associations with mood disorders and affective temperamental dimensions (namely cyclothymia), eating disorders characterized by overeating/binge-eating behaviors, ADHD-related executive dysfunctions, emotional dysregulation and motivational–addictive disturbances. With this review, we attempt to provide the clinician a synoptic, yet exhaustive, tool for a more conscious approach to that subset of this condition, which could be reasonably termed “psychiatric” obesity.

## 1. Introduction

Obesity is a complex disorder characterized by an increase in body fat mass and defined by a body mass index (BMI) greater than or equal to 30 kg/m^2^ [1]. Globally, its prevalence has reached pandemic proportions in the last decades, becoming a major concern for clinical and public health [2]. It is impressively estimated that about a third of the world population can be now classified as either overweight or obese [3]. The etiopathogenesis of obesity is multifactorial, implying both genetic predisposition and environmental factors, whose relative weight in the determinism of the condition can differ largely from case to case [1,2]. Currently available treatment strategies include dietary/behavioral, pharmacological and surgical approaches [4]. Nonetheless, the overall clinical outcome of obesity is relatively poor, mainly due to its resistance to treatment and high rate of relapse [1,4]. A great body of evidence shows that psychopathologic factors contribute extensively to the development and maintenance of this condition, thus lending intriguing insights to obesity medicine and representing possible targets for treatment and prevention [5,6].

## 2. A Bidirectional Relationship

Current evidence suggests that the relationship between obesity and psychopathology is of a bidirectional nature, since obesity has been repeatedly associated with manifold neuropsychic dysfunctions and, concurrently, varied subsets of psychiatric patients have consistently been shown to be burdened with an increased risk of developing obesity [7,8].

**Figure 1:**
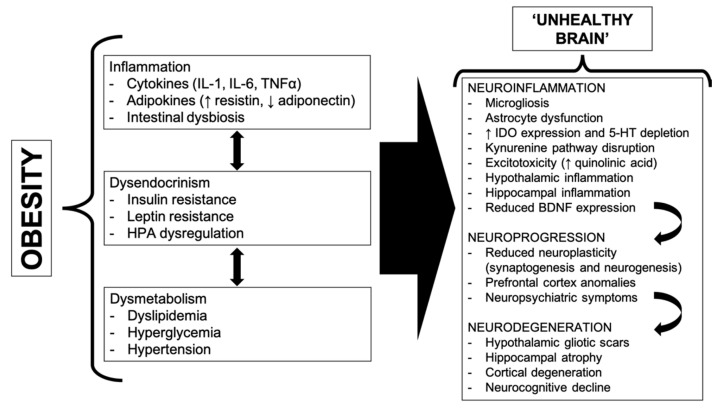
Theoretical model attempting to delineate some of the most significant pathobiological mechanisms, currently thought to explain the link between obesity and brain function. Acronyms—IL-1—interleukin 1; IL-6—interleukin 6; TNFα—tumor necrosis factor alpha; HPA—hypothalamus–pituitary–adrenal; IDO—indoleamine-2; 3-dioxygenase; 5-HT—5-hydoxytriptamine; BDNF—brain derived neurotrophic factor.

In order to provide a sound conceptual basis for our discussion, before we delve into the “psychopathology-to-obesity” pathway (which is the main topic of the present review) we will briefly expound the physiopathologic foundations of the “obesity-to-psychopathology” route (Figure 1). Obesity variably implies intermixed pathogenetic pathways, including sustained inflammation, endocrine disorders and metabolic dyshomeostasis, which influence and compound each other, possibly being, at once, both cause and effect. This complicated organismic perturbation is reflected on brain health, inducing a peculiar and regionalized inflammatory process, referred to as “neuroinflammation”. Neuroinflammation is characterized, among other features, by a functional (and, sometimes, histological) imbalance between microglial cells and astrocytes, the former overcoming the latter in many neurochemical processes, such as neurotransmitters metabolism and growth factors production. At length, plasticity processes (synaptic production and remodeling, neurogenesis) are impaired, especially in critical areas (hypothalamus, hippocampus and prefrontal cortex), a phenomenon called “neuroprogression”. Eventually, neurodegenerative processes can ensue, which involve, by definition, irreversible lesions (such as gliotic scars) and, more importantly, an appreciable loss of neural cells in both allocortical and isocortical areas. These structural alterations can partly explain why obesity predisposes to psychiatric disorders development (e.g., mood disorders) and provide a theoretical frame for the association between weight status and neuropsychic functions, such as cognitive/executive functions and emotion regulation. For a more thorough examination of the mechanisms mentioned above, see the recent reviews by Stapelberg et al. [9,10] and Shefer et al. [8].

Henceforward, we will deal with the “psychopathology-to-obesity” pathway, with the aim to outline the scope and possibilities of a “primarily psychiatric” subclass of patients with obesity. First, we will address the relationship between obesity and psychiatric disorders, specifically eating disorders, mood disorders and ADHD. In the following section, we will expound a possible placement of obesity-related psychopathology in the context of the temperamental framework of affective traits. Then, we will discuss the connection of obesity with executive dysfunctions, impulsivity and emotional dysregulation. After which, we will digress the possible role of “food addiction” in obesity. Finally, we will attempt to provide an interpretive and synthetic reflection, in the light of the literature we have reviewed.

## 3. Obesity as a Result of an Eating Disorder

The idea of viewing obesity—or at least a subset of it—as a result of an eating disorder (ED) has gained increasing acceptance in the last years, since subjects with obesity often display aberrant eating patterns, linked to emotional dysregulation or executive dysfunction and are prone to develop a “food addiction”, for which many authors have suggested a genetic basis of dopaminergic insufficiency in the mesolimbic pathway [11,12,13,14]. Obesity, in facts, is commonly accompanied by variable disorganization in eating habits and deficits in the regulatory mechanisms of appetite, reward and emotions [15,16,17,18]. For example, obesity is associated with leptin and insulin resistance, which results in an impaired capacity of sensing the nutritional status of body disrupts the hypothalamic control of energy homeostasis [19]. Additionally, obesity has recently been associated with anomalies in reward circuitry and the neural substrates of emotion, especially the amygdala, which in turn lead to a suprahomeostatic dysregulation of eating–feeding behavior [20].

Apart from probably implying a global eating regulation disorder in itself, obesity is often associated with canonical eating disorders, namely binge-eating disorder (BED) and bulimia nervosa (BN) [21]. BED is defined by a history of recurrent binge-eating episodes, during which the person eats unreasonably large amounts of food in a short time span while experiencing loss of control over this action, up to a point of uncomfortable or even painful, fullness. Such episodes usually occur in isolation, are not related to hunger or satiety and are followed by feelings of shame and guilt. What distinguishes BED from bulimia nervosa is the absence of compensatory purging behaviors, such as self-induced vomiting and inappropriate use of laxatives and diuretics [17,21].

The prevalence of BED in the general population ranges from 2% to 5% depending on the studies [22]. BED prevalence reaches variably higher proportions in the population of patients with obesity. More precisely, it seems that BED comorbidity correlates with the severity of weight excess, subjects with mild obesity (30 ≤ BMI < 35) showing comorbidity rates around 10%–15%, which rise up to 30% in treatment-seeking patients and even to 60% in those who eventually turn to bariatric surgery [22]. Most of patients with BN results in a stationary weight because of compensatory behaviors while a small proportion of them may present with obesity. A study conducted on a clinical sample of 1383 individuals with current eating disorders found that 87% of individuals with BED and 33% of individuals with BN was obese at some point in their lives [23].

There still remains uncertainty as to whether those who previously suffered from overweight/obesity are at a greater risk of developing an eating disorder later in life, and, vice versa, those who had a previous eating disorder are at higher risk of developing obesity. On one hand, there is evidence that having a history of a BMI ≥ 25 is not protective against developing restrictive eating habits and overt eating disorders during adolescence [24,25]. This possibility should be considered by clinicians, because, if neglected, it could lead to a detrimental diagnostic delay. On the other hand, a bit dated review of the literature suggests that only a small proportion (2%–10%) of patients with anorexia nervosa will gain excessive weight (BMI ≥ 25) during their lifetime [26].

A behavioral deviance strongly linked to BED is emotional eating, which is a form of pathologic eating wherein the person feels the urge to eat in response to stressful thoughts or situations, especially intense emotions with a negative valence [27]. There are three groups of theories explaining the relationship between emotions and eating: psychosomatic theories (as Kaplan and Kaplan’s [28] or Bruch’s [29]), which view overeating as a means of reducing stress and anxiety—typically in subjects with interoceptive, alexithymic and emotion-regulatory deficits; internal/external theories (as Schachter’s [30,31]), which interpret non-normative eating as the result of predominant nonhomeostatic, external cues over internal homeostatic signals in alimentary drives regulation; and restraint theories, which state that the self-control of people with obesity, always struggling to restrain their food intake, may be temporarily and explosively disrupted by stressful events [27,32]. Binge eating and emotional eating could be two sides of the same coin [33]. In fact, the most accepted theory explaining the psychopathologic basis of binge-eating is the affect regulation model, which postulates that binge-eating is a learned behavior acted with the purpose of coping with negative effects that the person is not able to manage otherwise [34]. Therefore, the nuclear element of BED-comorbid obesity would be emotional dysregulation, in terms of production of dysfunctional affective reactions, poor understanding of such reactions (alexithymia) and recourse to maladaptive strategies of coping (uncontrolled eating) [34]. Some authors even prompt that the reason for pathologic eating (emotion dysregulation) may be more important than the objective behavior (eating large amount of foods in little time) and subjective experiences (loss of control over eating) in the diagnosis of this disorder [35]. Executive dysfunctions, especially poor cortical inhibitory control and impulsivity are also associated with eating disorders characterized by loss of control (that is, the whole spectrum from binging/purging anorexia nervosa, through bulimia nervosa and binge-eating disorders) [36,37].

Besides all these conjectures, what must capture our attention is that BED-comorbid obesity is more challenging to manage and displays poorer outcomes, due to both psychopathologic and medical complications. BED patients with obesity actually have a significantly worse metabolic profile in comparison with non-BED patients, including higher fasting insulin levels, insulin resistance estimated through HOMA index, HbA1c (glycated hemoglobin), uric acid blood levels and visceral fat accumulation [38,39]. The association between BED, type 2 diabetes mellitus and non-alcoholic fatty liver disease seems to be especially strong, insofar as up to 25% of patients with type 2 diabetes and 23% of those with fatty liver disease have been reported to also have BED [40,41]. Patients with both obesity and BED display a heavier psychiatric burden compared with non-BED patients, and a great number of studies report higher levels of psychological and behavioral dysfunctions (mainly manifesting in the form of low self-esteem, negative self-evaluation, impulsivity, poor interoceptive awareness) and a broader presence of psychiatric disorders in patients with obesity who have ever engaged in binge-eating episodes [21,22]. It is estimated that patients, with a previous or current diagnosis of BED, have a lifetime proportion of psychiatric comorbidity approximating 80%, mainly attributable to mood, anxiety and substance use disorders [42].

## 4. Mood Disorders and Obesity

There is evidence that obesity and affective disorders represent reciprocal risk factors [43,44,45,46,47]. This mutual association is so strong and wide-ranging that some authors have begun to write about a “metabolic-mood syndrome” [48]. Epidemiological studies show that individuals with a history of a major depressive disorder (MDD) or BD are more likely to develop obesity than control population, and that patients with obesity are at increased risk of developing mood disorders, notably major depression [44]. According to a study from 2006, the lifetime prevalence of all mental disorders was higher in people with obesity than people without this condition. In particular, the prevalence of all mood disorders was 22% in subjects with obesity, in contrast with only 18.3% in subjects with a BMI < 30 (OR 1.27, CI 1.15–1.41). More specifically, the respective lifetime prevalences of MDD and BD were 18.6% and 2.8% in subjects whose BMI was equal to or higher than 30, and 16% and 1.9% in those who had a BMI lesser than 30 (OR 1.21 and 1.47, respectively) [49].

It has long been known that MDD is a risk factor for obesity. Obesity, in turn, is a risk factor for MDD, and there is a bidirectional pathophysiological bond between these conditions, so that we can speak of “obesity-associated depression” and “depression-associated obesity” [10]. In particular, a metanalysis of longitudinal studies concluded that patients with obesity had a 55% increased risk of developing MDD in their lifetime, while patients suffering from MDD have a 58% risk of developing obesity [50]. Since both conditions are highly and increasingly prevalent, investigating these reciprocal connections is of particular interest in many domains of healthcare. On one hand, “obesity-associated depression” is likely determined by two types of pathogenic determinants: a biologic determinant, consisting of perturbations in the psycho-immune-neuro-endocrine network (PINE network), such as chronic subinflammatory states, imbalance in the leptin-ghrelin system and leptin-ghrelin hypo-sensitivity, insulin resistance, gut dyspermeability, dysbiosis, endocrine and autonomic dysfunctions; and a psychological determinant, related to body image dissatisfaction, low self-esteem and consequent distress [10].

On the other hand, it may seem paradoxical to talk about a “depression associated obesity”, in consideration of the fact that classic melancholic depression is typically associated with hyporexia/hypophagia and loss of weight. Nevertheless, a depression-to-obesity causation can be purported in patients suffering from depressive illness with atypical features, i.e., a depression characterized by neuro-vegetative disturbances opposite to those of typical depression, such as hyperphagia and hypersomnia [10,51,52,53]. Depression with atypical features is much more common in women than in men (which could partly explain why the association between obesity and depression is reportedly stronger in women) [10,54] and usually develops on a basis of emotional dysregulation and interpersonal rejection hypersensitivity, both of which are also more common in females [51].

The psychopathologic ground of atypical depression extensively overlaps with that of BD type II, closely recalling the cyclothymic–anxious–sensitive matrix that characterizes the soft bipolar spectrum [51,55]. It is therefore not surprising that BD likewise increases the risk of overweight, obesity and pathologic eating, both in adults and adolescents [43,46,56]. More quantitatively, a cross-sectional study from 2011 found that BD significantly increased the risk of being obese (odds ratio (OR) = 1.65, 95% confidence interval (CI): 1.45–1.89, *p* < 0.001) [57]. Some psychotropic drugs used to treat BD can cause weight gain, but this association seems to hold true even in medication-naïve patients and after controlling the results for medication use [58,59]. Importantly, these conditions exert a mutual effect in worsening the prognosis of comorbid patients [46]. BD makes obesity more difficult to treat and psychotropic drugs used in its management can induce further weight gain, in particular atypical antipsychotics (their combination with mood stabilizers seems to be the most deleterious in this regard) [46]. Among antipsychotics, those associated with the greatest weight increase are clozapine and olanzapine, while among mood–stabilizers valproic acid seems to be the most obesogenic, although lithium salts are also frequently associated with weight gain [47]. Moreover, BD is commonly accompanied by eating disorders, in particular BED, which leads to a more severe form of obesity, both in terms of BMI and management difficulties [21,35,60]. Conversely, substance use disorder (SUD) seems to correlate with a lower BMI, prompting that drug and food addictions compete for the same reward circuits and tend to be mutually exclusive [57,61].

Mood disorders are also associated with various levels of dysexecutive features and emotional dysregulation, which could represent a further neuropsychological link between obesity and psychiatric disease. A meta-analysis from 2013 revealed a strong association between MDD and poor executive performances, including working memory, shifting and inhibition [62]. This association seems to persists, to a variable, but significant extent, after episodes remission and is not, in all probability, just a consequence of mood states [63,64]. Similarly, a recent meta-analysis has shown that individuals who ever had MDD exhibit poorer emotion regulation capabilities relative to subjects who have never been depressed [65].

In general, psychiatric comorbidity seems to compound the clinical outcomes of obesity and to undermine long-term weight loss achievement [47]. Obesity, in turn, complicates the clinical course of BD: bipolar patients with obesity reportedly have a higher frequency of mood episodes, shorter euthymic periods and are more resistant to lithium-based therapy compared with patients without obesity [46,66,67]. It has also been observed that obesity reduces the response of depressive disorders to antidepressant pharmacotherapy, but relevant results are less conclusive [68]. BD is frequently complicated by cardiovascular diseases, type 2 diabetes mellitus and metabolic syndrome, each of which bears a well-known risk relation with obesity [69,70]. Obesity is also associated with earlier cognitive decline (allegedly due to its above-outlined “encephalotoxic effect”), which can make BD management even more complicated [46,71].

Interestingly, there is oftentimes extensive phenomenological overlap between patients with obesity (especially if comorbid with BED) and those with BD as to many different features, such as temperamental aspects of mood instability and impulsivity, an inclination for erratic eating habits and “externalized” eating episodes, difficulty in respecting regular physical activity programs, disordered sleep patterns and possibly other imbrications [35,72].

## 5. ADHD and Obesity

Attention deficit and hyperactivity disorder (ADHD) is a common neurodevelopmental divergence characterized by inattention, hyperactivity and impulsivity as the cardinal triad. The association between obesity and ADHD, which has been systematically investigated for several years now, is seemingly critical for a better understanding of the psychopathologic dysfunctions that may drive the development of obesity [73,74,75]. Some authors have challenged the association between obesity and ADHD, but these results seem to be outshone by the overwhelming majority of favorable data [76,77,78]. Importantly, inasmuch as both conditions are very common, this association concerns a sizable portion of the care-seeking population [78]. The prevalence of ADHD in the population with a BMI ≥ 30 may be as high as 27.4%, whereas in the general population it stands at about 3%–4% [74]. In turn, adults and children with ADHD have, respectively, a 70% and 40% increased risk of developing obesity [78]. The association is stronger in adolescents and adults than in children, perhaps because ADHD’s effect on obesity is gradual and does not fully emerge until adolescence [79].

Many heterogeneous pathogenetic pathways have been proposed to explain the connection between these two conditions. Structurally, obesity and ADHD have been hypothesized to share common underlying neurobiological and neuropsychological abnormalities such as dysfunctions in brain reward pathways, emotion regulation processes and executive functions [80]. Adults with ADHD often exhibit unhealthy eating habits and feeding anomalies [75]. For instance, difficulty in planning, as a consequence of inattentiveness, often translates into skipping meals and eating between meals or at inappropriate times (night eating) [81]. Emotional dysfunction, which is a common finding in subjects with ADHD, can lead to experiencing emotional hunger/eating and this can progressively generate episodes of full-blown binge-eating, allegedly on the basis of a typical ADHD-linked impulsive trait [82,83]. Children and adults with ADHD usually suffer from short sleep duration due to initial insomnia, which has been ascribed to a delayed onset of melatonin [84]. Short sleep duration has repeatedly been associated with the development of obesity and has therefore been suggested as an additional connection mechanism [85]. Youth with ADHD, both medicated and unmedicated, seem to be more unlikely to meet the recommended physical activity levels (e.g., 150 min per week) than their non-ADHD peers [86,87].

Treating ADHD in childhood has been shown to reduce the risk of developing obesity in adulthood [78]. ADHD possibly represents a major cause of treatment failure in adults with severe obesity and treating ADHD with methylphenidate optimizes clinical outcomes in patients with obesity and ADHD [88]. Weight reduction cannot be ascribed solely to the anorectic effect of methylphenidate, as this effect vanishes in a few weeks, while patients keep on losing weight or maintain the loss much longer [88,89]. Remission of the cardinal symptoms of ADHD results in more efficient executive functions, which probably empowers patients to better comply with diet and take on enduring changes in their lifestyle. Such statement is corroborated by experiments that showed the beneficial effect of selective executive function training on weight loss in children with obesity and ADHD [90].

## 6. An Affective-Temperamental Framework for Obesity

Affective temperaments are constitutionally determined affective dispositions, embodied by rather stereotypical collections of stable emotion-related traits and subserving evolutionary purposes [91]. According to the Temperament Evaluation of Memphis, Pisa, Paris and San Diego (TEMPS) scales, five affective temperaments are described and sought: hyperthymic, cyclothymic, dysthymic, irritable and anxious [92]. Temperaments exist a priori with respect to experience and, mingling with experience-derived, concept-based character traits, contribute to the molding of personality [93]. Despite not constituting a disorder in itself, the presence of a pronounced temperamental dimension predisposes to the development of full-blown mood episodes and seemingly modifies their specific phenomenology (the so-called “pathoplastic” effect) [94,95].

Given its frequent comorbidity with bipolar spectrum disorders, obesity (notably BED-comorbid obesity) may be associated with marked temperamental dimensions, cyclothymic and anxious in particular [35,96,97,98]. Cyclothymic temperament is often characterized by sharp affective lability and reactivity, admixed with interpersonal sensitivity, poor self-awareness, impulsiveness and “sensation-seeking” self-stimulatory trends [51,72,99]. Considering the hypothesis that binge-eating attacks could be a dysfunctional regulatory strategy to cope with negative affects—or at least an impulsion triggered by adverse emotionality—cyclothymic–anxious–sensitive subjects could be reasonably more prone to develop binge-eating behaviors and related disorders (anorexia nervosa bingeing–purging subtype, bulimia nervosa and BED) [99,100].

There are only a handful of studies addressing the issue of obesity-temperament association and results are not always consistent. Although a cross-sectional study from 2009 showed higher scores in cyclothymic, anxious and irritable dimensions among bariatric patients, a more recent study found a negative correlation between the BMI and cyclothymic temperament, while reporting a positive correlation with the hyperthymic temperament [101,102]. Another cross-sectional study from 2004 found that eating disorders characterized by binge-eating episodes (i.e., all, but restrictive anorexia nervosa) are associated with cyclothymic, dysthymic and hyperthymic temperamental dimensions [103]. A more recent study found that patients with bingeing–purging type of anorexia nervosa had greater cyclothymic and anxious dimensions compared to those with the restricting type [104]. A Tunisian study found a significant association between all-type eating disorders and cyclothymic temperamental dimension [105].

In the light of the foregoing, subthreshold temperamental features seem to be more deeply related to binge-eating syndromes than to obesity in itself, yet still further investigation is necessary to substantiate this hypothesis.

## 7. Executive Dysfunctions, Impulsivity and Emotional Dysregulation Are Involved in Obesogenesis

Executive functions can be defined as those “high level” activities, mainly worked out by the prefrontal cortex, which allow human beings to produce complex, self-regulatory, goal-oriented thoughts and behaviors through modulation of “lower level” subcortical responses [106]. Obesity is deemed to be associated by reciprocal causation with several possible deficiencies in these functions [107]. Executive dysfunctions, such as poor inhibitory control and an increased delay discounting rate, could lead to detrimental eating patterns, whether in quantitative terms (overeating, gorging, grazing, snacking) or in qualitative terms (craving for palatable, high-sugar, high-fat foodstuffs) and disincline the subject to undertake lasting effective changes in diet and lifestyle. Coherently, cognitive training approaches have recently demonstrated some effectiveness in facilitating weight loss and reducing unhealthy behaviors [108,109]. In addition, pretreatment cognitive functions have been suggested as a prognostic index for the success of weight loss programs and the outcome of bariatric surgery [110,111], although data on this association are still heterogeneous [112,113]. Obesity, in turn, is thought to biologically promote neuroprogression/-degeneration, and recent studies show that BMI reduction improves cognitive function and memory performances in patients with obesity, thus supporting the hypothesis of some obesity-driven brain dysfunctions [114,115,116].

Impulsivity is a psychopathologic trait closely related to executive functions (especially inhibitory control), defined by Moeller, Barratt, Dougherty, Schmitz and Swann (2001) as the “predisposition toward rapid, unplanned reactions to internal or external stimuli without regard to the negative consequences of these reactions to the impulsive individuals or to others” [117]. There are two main subtraits of impulsivity: rapid response impulsivity (or “motor impulsiveness” in Barrattian terminology) which is the tendency to react rapidly to internal or external stimuli, without adequate forethought; and reward delay or choice or decisional impulsivity (much similar to the “nonplanning” impulsiveness of Barratt’s and Eysenck’s scales) which is defined by the intolerance for reward delay and the increased rates of temporal discounting [117,118,119]. Both studies using self-report questionnaires and those using behavioral tasks support the concept that obesity and eating disorders are associated with various levels and types of impulsive subtraits, which are supposedly accounted for by a deficient top–down cognitive inhibitory control over prepotent responses and subsequent imbalance between impulsive and reflective systems [111,120]. In particular, the population with obesity as a whole seems to be more impulsive and more sensitive to reward than normal weight control subjects, and patients with BED seem to exhibit higher levels of “rapid response” or “motor” impulsivity [118,120,121]. For example, a recent cross-sectional study, which used BIS-11 scale to measure impulsivity in a sample of 11,929 men and 39,114 women from the general population, found a strong association with high levels of impulsivity (BIS-11 > 71) in men with class III obesity (OR 3.57, *p* = 0.0028) and weaker, but significant associations with lower classes of obesity and in women [120]. Another more dated study found that women with obesity and BED exhibit significantly greater levels in the motor impulsivity subscale of BIS-11 scale, relative to women with obesity, but without BED (F = 3.3, *p* = 0.05) [118].

Motor impulsiveness has been found to be associated also with the other eating disorders implying a binge/loss of control eating behavior (bulimia nervosa and anorexia nervosa, bingeing–purging type), differentiating bingeing patients both from healthy controls and from anorexia nervosa, restrictive type [122]. It is then reasonable to think that “nonplanning” impulsivity, in particular reward hypersensitivity and intolerance for deferred gratification, contributes to steer dietary choices of patients with obesity towards highly caloric and palatable (although unhealthy) foodstuffs, and to incapacitate them from procrastinating today’s pleasure so as to obtain a greater good, viz. psychophysical health, in the future [123]. Motor impulsivity may add a sprinkling of explosiveness to these behavioral tendencies, leading to episodes of full-fledged binge-eating [124].

As hinted at above, aspects of emotional dysregulation seem to be crucial both in obesity and eating disorders psychopathology, and it is not always easy to differentiate which emotional dysfunctions are related to obesity as a whole and which are specific to BED-comorbid obesity [125]. Possible emotional dysfunctions can arise in every step of the emotional flow, from affective over-reactivity or lability, through alexithymia (affective unawareness or illiteracy), to regulatory strategies (that is, emotional regulation in the strict sense) [125]. In particular, extant literature suggests that patients with obesity and BED are globally less skilled in managing their affective processes, both in terms of emotions acknowledgment (alexithymia) and regulation [33,126]. When compared to obesity-BED comorbid patients, subjects with obesity, but without BED show similar, although milder, alexithymic defects in emotional processing, along with a tendency towards external-oriented thinking (EOT) style, but less difficulties with keeping a goal-oriented behavior during stressful experiences [125,127]. Anyway, aside from past and current speculations over their psychopathologic motivations, what is quite ascertained is that binge-eating crises are very often preceded by the experience of negative effects, and that conditions involving mood lability/reactivity and poor emotion-regulatory capabilities predispose to abnormal eating behaviors [96,126,128,129].

## 8. A Motivational Contribution: Eating and Food Addiction

A relatively new idea is that some kinds of food, especially calorie-rich, fatty and sugary foods, can become addictive in susceptible individuals [130]. More precisely, the food addiction hypothesis can be conceptualized as a twofold postulate: on one hand, it states that there exist certain individuals (often, but not always, suffering from obesity) who develop a behavioral syndrome, characterized by craving for food and loss of control over eating in spite of its known detrimental health effects, which is quite similar to classic behavioral addiction (eating addiction); on the other hand, it claims that some specific foodstuffs, namely high calorie sugar/fat-rich foods, can activate the reward system as classic addictive drugs do (food addiction) [16,131]. Although a recent systematic review concludes in its favor [132], it is necessary to point out that the “food addiction” construct is still much controversial and represents a thorny subject in the current scientific debate. So, caution is needed when addressing this problem, given that its precise boundaries are not well defined, especially regarding its similarities and differences, than other, more established, forms of addiction. [133].

The prevalence of food addiction in the population with obesity is highly variable (from 7.7% to 47%), possibly depending on weight excess severity and clinical context, but seems to be consistently higher in females and older people [134,135,136]. According to an Italian cross-sectional study, food addiction, measured by the Yale Food Addiction scale (YFAS), was found in nearly 34% of patients seeking treatment for obesity, while a recent German cross-sectional study reported a 27% prevalence of food addiction in bariatric surgery candidates [137,138]. Another German cross-sectional study found a nonlinear correlation between food addiction and BMI, with food addiction being most prevalent in the underweight (15%) and obesity (17.2%) categories [139]. This “*U-*shaped” correlation, alongside the evidence of an association between food addiction symptoms and non-obesogenic eating disorders (anorexia nervosa and bulimia nervosa), invites to view food addiction as a trans-nosographic construct, more connected to a ground eating-related psychopathology than to raw BMI in itself [140]. Anyway, the higher prevalence of food addiction in the underweight category may be partly explained by their subjective perception of “excessive eating”, even when they eat a meagre amount of food, and could call into question the reliability of YFAS in underweight patients with restrictive eating behaviors.

If patients with emotionally based eating behaviors have multitiered affective dysfunctions which result in eating impulsions (forced eating before thinking of eating), some other patients with obesity may exhibit eating compulsions (forced eating after thinking of eating) on a dysmotivational basis [141]. Anyway, since there is often much overlap between impulsive and compulsive behavioral deviances, it is not always feasible to distinguish them. In particular, it seems that a large portion (40%–70%) of BED patients experiences addictive-motivational symptoms, and this subset seems to be burdened with a graver psychopathology, both in terms of psychiatric comorbidity, emotional dysfunctions and impulsivity, thus corroborating the concept of an ample imbrication between compulsive and impulsive components in bulimic behaviors [131,142]. An evocative (and a bit disquieting) finding, concerning the role of food addiction in obesogenesis, is the longitudinal observation that post-bariatric patients are reportedly at high risk of substituting their previous food compulsion/impulsion with substances or alcohol abuse, a phenomenon known as “addiction shift or transfer” [143,144]. In spite of all these arguments, the staunchest piece of evidence in favor of the “food addiction hypothesis” still comes from neurobiologic studies of the reward system in animals and humans [132].

## 9. Is There a Common Thread?

**Figure 2:**
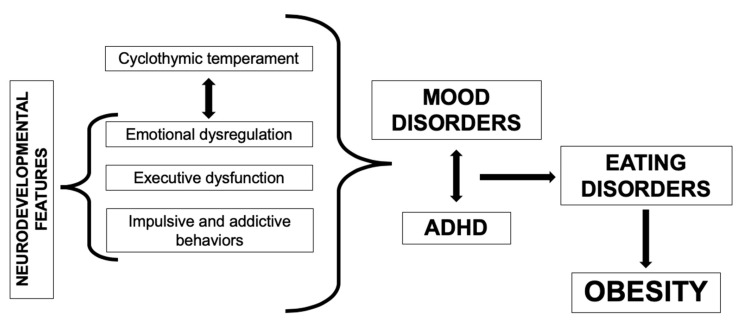
Obesity-related neuropsychiatric syndromes display a substantial overlap or homology, suggesting the existence of a common psychopathologic ground for the emergence of maladaptive eating behaviors, which can lead to obesity in the long run.

In a substantial proportion of cases, obesity with emotional eating behaviors and bipolar spectrum disorders may be conceptualized as manifestations of a common psychopathologic ground, represented by a cyclothymic-anxious-sensitive temperamental dimension. Such affective disposition may explain many phenomenological features of BED patients, namely emotional dysfunction (affective instability and reactivity) and dysregulation (alexithymia, avoidance, suppression, lack of reappraisal), impulsivity and motivational disturbances [72]. Many of these behavioral–affective problems, along with other dysexecutive symptoms, are also part and parcel of ADHD’s psychopathology, suggesting that a shortage of executive (“cold executive”) and motivational-emotional (“hot executive”) resources underlies a major subset of non-normative eating behaviors, in which food intake is subverted by supra-homeostatic, affective-hedonic mechanisms that overpower its fine-tuned, homeostatic–hypothalamic regulation (Figure 2) [75].

**Figure 3:**
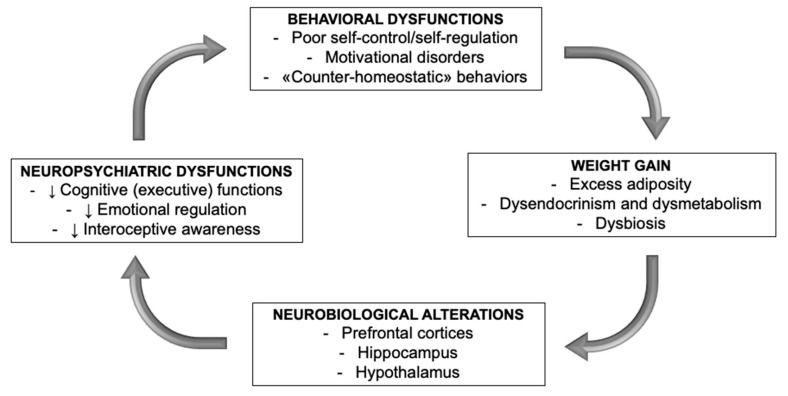
Obesity-associated neuropsychiatric dysfunctions, outlined elsewhere in the text, entail inadequate self-control and unhealthy behaviors, which lead to excessive, disordered eating and weight gain. In its turn, weight gain is accompanied by a whole complexity of pathobiological alterations, which aggravate the neuropsychiatric dysfunctions by engendering a neurofunctional and, subsequently, neuroanatomic damage.

“Psychiatric obesity” appears to be ruinously linked to brain performances by a Galenic *circulus vitiosus*, where executive and emotional dysfunctions make the subject helpless before the temptations of our obesogenic environment obesity-associated neuroendocrine disruptions further impair their capability to hold the reins of vice and interrupt their unmerciful progression towards an unbreakable, irremediable psycho–physio–pathologic trap (Figure 3) [8].

## 10. Limitations

One of the most important limitations of our narrative review is certainly the lack of a systematic analysis of available data. Another relevant limitation is the asymmetry in the examination of the bidirectional relationship between obesity and psychopathology, with greater attention paid to the “psychopathology-to-obesity pathway”. On the other hand, the main strength of this review is to provide a theoretical overview of the recent literature related to the association of obesity with psychiatric disorders and neuropsychiatric features, especially in the affective-emotional and the cognitive-executive dimensions. In fact, to our best knowledge, there is a wealth of reviews concerning the effects of obesity-related endocrino–metabolic and immunological derangements on neuropsychic functioning, whereas we found only a paucity of literature presenting an overall view of psychopathologic influences on obesity development and perpetuation.

## 11. Conclusions

Obesity is associated with a growing number of neuropsychiatric problems, indubitably involved in the pathogenesis of this condition. An increasing body of evidence is recently showing that the immune–metabolic–endocrine alterations, notoriously associated with excess body weight/adiposity, impair the morpho-functional integrity of the brain, thus possibly leading to detrimental neurobiologic processes and behavioral deviances. Concomitantly, in a more clinical perspective, obesity exhibits complex associations with mood disorders, affective temperamental dimensions (especially cyclothymia), eating disorders and ADHD-related executive, emotional and motivational dysfunctions. To date, addressing the possible relationships between obesity and these psychiatric aspects still looks largely like a leap into the unknown. Preliminarily, the evidence hereinbefore gathered seems to point towards a new clinical approach to the patient suffering from obesity, who should be subtyped as “psychiatric” and “non-psychiatric”, on the basis of the presence or absence, of certain risk factors, whether they be overt neuropsychiatric diagnoses (bipolar spectrum disorders, eating disorders and ADHD, in particular) or just subsyndromal neuropsychological frailties (especially emotional dysregulation and executive dysfunction). We suggest that managing the psychopathologic ground of certain obesity subsets may provide a prognostic advantage to conventional strategies, either conservative or invasive. Further research is needed, with the aim to advance our knowledge of the psychopathologic factors involved in the development and maintenance of this condition and to eventually device a more precise nosography within “psychiatric obesity”, which should allow us to advisedly weigh up the actual profitability of a baseline psychopathologic assessment of all treatment-seeking patients with obesity.

## References

[1] Heymsfield S.B., Wadden T.A. (2017). Mechanisms, Pathophysiology, and Management of Obesity. N. Engl. J. Med..

[2] Blüher M. (2019). Obesity: Global epidemiology and pathogenesis. Nat. Rev. Endocrinol..

[3] Chooi Y.C., Ding C., Magkos F. (2019). The epidemiology of obesity. Metabolism.

[4] Durrer Schutz D., Busetto L., Dicker D., Farpour-Lambert N., Pryke R., Toplak H., Widmer D., Yumuk V., Schutz Y. (2019). European Practical and Patient-Centred Guidelines for Adult Obesity Management in Primary Care. Obes. Facts.

[5] Carpiniello B., Pinna F., Pillai G., Nonnoi V., Pisano E., Corrias S., Orru M.G., Orru W., Velluzzi F., Loviselli A. (2009). Obesity and psychopathology. A study of psychiatric comorbidity among patients attending a specialist obesity unit. Epidemiol. Psichiatr. Soc..

[6] Lopresti A.L., Drummond P.D. (2013). Obesity and psychiatric disorders: Commonalities in dysregulated biological pathways and their implications for treatment. Prog. Neuro-Psychopharmacol. Biol. Psychiatry.

[7] Castanon N., Lasselin J., Capuron L. (2014). Neuropsychiatric comorbidity in obesity: Role of inflammatory processes. Front. Endocrinol..

[8] Shefer G., Marcus Y., Stern N. (2013). Is obesity a brain disease?. Neurosci. Biobehav. Rev..

[9] Stapelberg N.J.C., Neumann D., Shum D., Mcconnell H., Hamilton-Craig I. (2015). From Physiome to Pathome: A Systems Biology Model of Major Depressive Disorder and the Psycho-Immune-Neuroendocrine Network. Curr. Psychiatry Rev..

[10] Patist C.M., Stapelberg N.J.C., Du Toit E.F., Headrick J.P. (2018). The brain-adipocyte-gut network: Linking obesity and depression subtypes. Cogn. Affect. Behav. Neurosci..

[11] Jebb S.A., Prentice A.M. (1995). Is obesity an eating disorder?. Proc. Nutr. Soc..

[12] Jemma D.A.Y., Ternouth A., Collier D.A. (2009). Eating disorders and obesity: Two sides of the same coin?. Epidemiol. Psichiatr. Soc..

[13] Raman J., Smith E., Hay P. (2013). The clinical obesity maintenance model: An integration of psychological constructs including mood, emotional regulation, disordered overeating, habitual cluster behaviours, health literacy and cognitive function. J. Obes..

[14] Blum K., Thanos P.K., Gold M.S. (2014). Dopamine and glucose, obesity and reward deficiency syndrome. Front. Psychol..

[15] De Velasco R.M., Barbudo E., Pérez-Templado J., Silveira B., Quintero J. (2015). Review of the association between obesity and ADHD. Actas Esp. Psiquiatr..

[16] Ziauddeen H., Alonso-Alonso M., Hill J.O., Kelley M., Khan N.A. (2015). Obesity and the neurocognitive basis of food reward and the control of intake. Adv. Nutr..

[17] McCuen-Wurst C., Ruggieri M., Allison K.C. (2018). Disordered eating and obesity: Associations between binge-eating disorder, night-eating syndrome, and weight-related comorbidities. Ann. N. Y. Acad. Sci..

[18] Hayes J.F., Fitzsimmons-Craft E.E., Karam A.M., Jakubiak J., Brown M.L., Wilfley D.E. (2018). Disordered Eating Attitudes and Behaviors in Youth with Overweight and Obesity: Implications for Treatment. Curr. Obes. Rep..

[19] Könner A.C., Brüning J.C. (2012). Selective Insulin and Leptin Resistance in Metabolic Disorders. Cell Metab..

[20] Farr O.M., Li C.-S.R., Mantzoros C.S. (2016). Central nervous system regulation of eating: Insights from human brain imaging. Metabolism.

[21] da Luz F.Q., Hay P., Touyz S., Sainsbury A. (2018). Obesity with Comorbid Eating Disorders: Associated Health Risks and Treatment Approaches. Nutrients.

[22] Vaidya V., Malik A. (2008). Eating disorders related to obesity. Therapy.

[23] Villarejo C., Fernández-Aranda F., Jiménez-Murcia S., Peñas-Lledó E., Granero R., Penelo E., Tinahones F.J., Sancho C., Vilarrasa N., Montserrat-Gil De Bernabé M. (2012). Lifetime obesity in patients with eating disorders: Increasing prevalence, clinical and personality correlates. Eur. Eat. Disord. Rev..

[24] Lebow J., Sim L.A., Kransdorf L.N. (2015). Prevalence of a history of overweight and obesity in adolescents with restrictive eating disorders. J. Adolesc. Health Off. Publ. Soc. Adolesc. Med..

[25] Koritar P., Pinzon V.D., Barros C., Cobelo A., Fleitlich-Bilyk B. (2014). Anorexia nervosa: Differences and similarities between adolescents with and without a history of obesity. Rev. Mex. Trastor. Aliment..

[26] Herzog D.B., Keller M.B., Lavori P.W. (1988). Outcome in anorexia nervosa and bulimia nervosa. A review of the literature. J. Nerv. Ment. Dis..

[27] Van Strien T. (2018). Causes of Emotional Eating and Matched Treatment of Obesity. Curr. Diab. Rep..

[28] Kaplan H.I., Kaplan H.S. (1957). The psychosomatic concept of obesity. J. Nerv. Ment. Dis..

[29] Callaway W. (1973). Eating Disorders: Obesity, Anorexia Nervosa, and the Person Within. JAMA.

[30] Schachter S. (1968). Obesity and eating. Internal and external cues differentially affect the eating behavior of obese and normal subjects. Science.

[31] Schachter S., Goldman R., Gordon A. (1968). Effects of fear, food deprivation, and obesity on eating. J. Pers. Soc. Psychol..

[32] Canetti L., Bachar E., Berry E.M. (2002). Food and emotion. Behav. Processes.

[33] Gianini L.M., White M.A., Masheb R.M. (2013). Eating pathology, emotion regulation, and emotional overeating in obese adults with binge eating disorder. Eat. Behav..

[34] Dingemans A., Danner U., Parks M. (2017). Emotion Regulation in Binge Eating Disorder: A Review. Nutrients.

[35] Segura-Garcia C., Caroleo M., Rania M., Barbuto E., Sinopoli F., Aloi M., Arturi F., De Fazio P. (2017). Binge Eating Disorder and Bipolar Spectrum disorders in obesity: Psychopathological and eating behaviors differences according to comorbidities. J. Affect. Disord..

[36] Blume M., Schmidt R., Hilbert A. (2018). Executive Functioning in Obesity, Food Addiction, and Binge-Eating Disorder. Nutrients.

[37] Tamiya H., Ouchi A., Chen R., Miyazawa S., Akimoto Y., Kaneda Y., Sora I. (2018). Neurocognitive Impairments Are More Severe in the Binge-Eating/Purging Anorexia Nervosa Subtype Than in the Restricting Subtype. Front. Psychiatry.

[38] Succurro E., Segura-Garcia C., Ruffo M., Caroleo M., Rania M., Aloi M., de Fazio P., Sesti G., Arturi F. (2015). Obese Patients With a Binge Eating Disorder Have an Unfavorable Metabolic and Inflammatory Profile. Medicine.

[39] Wassenaar E., Friedman J., Mehler P.S. (2019). Medical Complications of Binge Eating Disorder. Psychiatr. Clin. N. Am..

[40] Raevuori A., Suokas J., Haukka J., Gissler M., Linna M., Grainger M., Suvisaari J. (2015). Highly increased risk of type 2 diabetes in patients with binge eating disorder and bulimia nervosa. Int. J. Eat. Disord..

[41] Zhang J., Abbasi O., Malevanchik L., Mohan N., Denicola R., Tarangelo N., Marzio D.H.-D. (2017). Pilot study of the prevalence of binge eating disorder in non-alcoholic fatty liver disease patients. Ann. Gastroenterol..

[42] Kessler R.C., Berglund P.A., Chiu W.T., Deitz A.C., Hudson J.I., Shahly V., Aguilar-Gaxiola S., Alonso J., Angermeyer M.C., Benjet C. (2013). The prevalence and correlates of binge eating disorder in the World Health Organization World Mental Health Surveys. Biol. Psychiatry.

[43] McElroy S.L., Kotwal R., Malhotra S., Nelson E.B., Keck P.E., Nemeroff C.B. (2004). Are mood disorders and obesity related? A review for the mental health professional. J. Clin. Psychiatry.

[44] Gadalla T.M. (2009). Association of obesity with mood and anxiety disorders in the adult general population. Chronic Dis. Can..

[45] Soczynska J.K., Kennedy S.H., Woldeyohannes H.O., Liauw S.S., Alsuwaidan M., Yim C.Y., McIntyre R.S. (2011). Mood disorders and obesity: Understanding inflammation as a pathophysiological nexus. Neuromol. Med..

[46] McElroy S.L., Keck P.E.J. (2012). Obesity in bipolar disorder: An overview. Curr. Psychiatry Rep..

[47] McElroy S.L., Guerdjikova A.I., Mori N., Keck P.E. (2016). Managing comorbid obesity and depression through clinical pharmacotherapies. Expert Opin. Pharmacother..

[48] Mansur R.B., Brietzke E., McIntyre R.S. (2015). Is there a “metabolic-mood syndrome”? A review of the relationship between obesity and mood disorders. Neurosci. Biobehav. Rev..

[49] Simon G.E., Von Korff M., Saunders K., Miglioretti D.L., Crane P.K., van Belle G., Kessler R.C. (2006). Association Between Obesity and Psychiatric Disorders in the US Adult Population. Arch. Gen. Psychiatry.

[50] Luppino F.S., de Wit L.M., Bouvy P.F., Stijnen T., Cuijpers P., Penninx B.W.J.H., Zitman F.G. (2010). Overweight, Obesity, and Depression: A Systematic Review and Meta-analysis of Longitudinal Studies. Arch. Gen. Psychiatry.

[51] Perugi G., Fornaro M., Akiskal H.S. (2011). Are atypical depression, borderline personality disorder and bipolar II disorder overlapping manifestations of a common cyclothymic diathesis?. World Psychiatry Off. J. World Psychiatr. Assoc..

[52] Lasserre A.M., Glaus J., Vandeleur C.L., Marques-Vidal P., Vaucher J., Bastardot F., Waeber G., Vollenweider P., Preisig M. (2014). Depression with atypical features and increase in obesity, body mass index, waist circumference, and fat mass: A prospective, population-based study. JAMA Psychiatry.

[53] Lojko D., Buzuk G., Owecki M., Ruchala M., Rybakowski J.K. (2015). Atypical features in depression: Association with obesity and bipolar disorder. J. Affect. Disord..

[54] Pratt L.A., Brody D.J. (2014). Depression and Obesity in the U.S. Adult Household Population, 2005–2010.

[55] Perugi G., Akiskal H.S., Lattanzi L., Cecconi D., Mastrocinque C., Patronelli A., Vignoli S., Bemi E. (1998). The high prevalence of “soft” bipolar (II) features in atypical depression. Compr. Psychiatry.

[56] Goldstein B.I., Birmaher B., Axelson D.A., Goldstein T.R., Esposito-Smythers C., Strober M.A., Hunt J., Leonard H., Gill M.K., Iyengar S. (2008). Preliminary findings regarding overweight and obesity in pediatric bipolar disorder. J. Clin. Psychiatry.

[57] Goldstein B.I., Liu S.-M., Zivkovic N., Schaffer A., Chien L.-C., Blanco C. (2011). The burden of obesity among adults with bipolar disorder in the United States. Bipolar Disord..

[58] Petry N.M., Barry D., Pietrzak R.H., Wagner J.A. (2008). Overweight and obesity are associated with psychiatric disorders: Results from the National Epidemiologic Survey on Alcohol and Related Conditions. Psychosom. Med..

[59] Maina G., Salvi V., Vitalucci A., D’Ambrosio V., Bogetto F. (2008). Prevalence and correlates of overweight in drug-naive patients with bipolar disorder. J. Affect. Disord..

[60] Fornaro M., Perugi G., Gabrielli F., Prestia D., Mattei C., Vinciguerra V., Fornaro P. (2010). Lifetime co-morbidity with different subtypes of eating disorders in 148 females with bipolar disorders. J. Affect. Disord..

[61] McIntyre R.S., McElroy S.L., Konarski J.Z., Soczynska J.K., Bottas A., Castel S., Wilkins K., Kennedy S.H. (2007). Substance use disorders and overweight/obesity in bipolar I disorder: Preliminary evidence for competing addictions. J. Clin. Psychiatry.

[62] Snyder H.R. (2013). Major depressive disorder is associated with broad impairments on neuropsychological measures of executive function: A meta-analysis and review. Psychol. Bull..

[63] Woo Y.S., Rosenblat J.D., Kakar R., Bahk W.-M., McIntyre R.S. (2016). Cognitive Deficits as a Mediator of Poor Occupational Function in Remitted Major Depressive Disorder Patients. Clin. Psychopharmacol. Neurosci..

[64] Paelecke-Habermann Y., Pohl J., Leplow B. (2005). Attention and executive functions in remitted major depression patients. J. Affect. Disord..

[65] Visted E., Vøllestad J., Nielsen M.B., Schanche E. (2018). Emotion Regulation in Current and Remitted Depression: A Systematic Review and Meta-Analysis. Front. Psychol..

[66] Fagiolini A., Kupfer D.J., Houck P.R., Novick D.M., Frank E. (2003). Obesity as a Correlate of Outcome in Patients With Bipolar I Disorder. Am. J. Psychiatry.

[67] Kemp D.E., Gao K., Chan P.K., Ganocy S.J., Findling R.L., Calabrese J.R. (2010). Medical comorbidity in bipolar disorder: Relationship between illnesses of the endocrine/metabolic system and treatment outcome. Bipolar Disord..

[68] Woo Y.S., Seo H.-J., McIntyre R.S., Bahk W.-M. (2016). Obesity and Its Potential Effects on Antidepressant Treatment Outcomes in Patients with Depressive Disorders: A Literature Review. Int. J. Mol. Sci..

[69] Kilbourne A.M., Cornelius J.R., Han X., Pincus H.A., Shad M., Salloum I., Conigliaro J., Haas G.L. (2004). Burden of general medical conditions among individuals with bipolar disorder. Bipolar Disord..

[70] Goldstein B.I., Schaffer A., Wang S., Blanco C. (2015). Excessive and premature new-onset cardiovascular disease among adults with bipolar disorder in the US NESARC cohort. J. Clin. Psychiatry.

[71] Yim C.Y., Soczynska J.K., Kennedy S.H., Woldeyohannes H.O., Brietzke E., McIntyre R.S. (2012). The effect of overweight/obesity on cognitive function in euthymic individuals with bipolar disorder. Eur. Psychiatry.

[72] Perugi G., Akiskal H.S. (2002). The soft bipolar spectrum redefined: Focus on the cyclothymic, anxious-sensitive, impulse-dyscontrol, and binge-eating connection in bipolar II and related conditions. Psychiatr. Clin. N. Am..

[73] Sandra Kooij J.J. (2016). ADHD and obesity. Am. J. Psychiatry.

[74] Altfas J.R. (2002). Prevalence of attention deficit/hyperactivity disorder among adults in obesity treatment. BMC Psychiatry.

[75] Cortese S. (2019). The Association between ADHD and Obesity: Intriguing, Progressively More Investigated, but Still Puzzling. Brain Sci..

[76] Dubnov-Raz G., Perry A., Berger I. (2011). Body mass index of children with attention-deficit/hyperactivity disorder. J. Child Neurol..

[77] Cortese S., Tessari L. (2017). Attention-Deficit/Hyperactivity Disorder (ADHD) and Obesity: Update 2016. Curr. Psychiatry Rep..

[78] Cortese S., Moreira-Maia C.R., St. Fleur D., Morcillo-Peñalver C., Rohde L.A., Faraone S.V. (2016). Association Between ADHD and Obesity: A Systematic Review and Meta-Analysis. Am. J. Psychiatry.

[79] Nigg J.T., Johnstone J.M., Musser E.D., Long H.G., Willoughby M.T., Shannon J. (2016). Attention-deficit/hyperactivity disorder (ADHD) and being overweight/obesity: New data and meta-analysis. Clin. Psychol. Rev..

[80] Seymour K.E., Reinblatt S.P., Benson L., Carnell S., Sciences B. (2016). Overlapping neurobehavioral circuits in ADHD, obesity, and binge eating: Evidence from Neuroimaging Research. CNS Spectr..

[81] Hanc T., Cortese S. (2018). Attention deficit/hyperactivity-disorder and obesity: A review and model of current hypotheses explaining their comorbidity. Neurosci. Biobehav. Rev..

[82] Hanson J.A., Phillips L.N., Hughes S.M., Corson K. (2019). Attention-deficit hyperactivity disorder symptomatology, binge eating disorder symptomatology, and body mass index among college students. J. Am. Coll. Health.

[83] Egbert A.H., Wilfley D.E., Eddy K.T., Boutelle K.N., Zucker N., Peterson C.B., Celio Doyle A., Le Grange D., Goldschmidt A.B. (2018). Attention-Deficit/Hyperactivity Disorder Symptoms Are Associated with Overeating with and without Loss of Control in Youth with Overweight/Obesity. Child. Obes..

[84] Van Veen M.M., Kooij J.J.S., Boonstra A.M., Gordijn M.C.M., Van Someren E.J.W. (2010). Delayed Circadian Rhythm in Adults with Attention-Deficit/Hyperactivity Disorder and Chronic Sleep-Onset Insomnia. Biol. Psychiatry.

[85] Vogel S.W.N., Bijlenga D., Tanke M., Bron T.I., van der Heijden K.B., Swaab H., Beekman A.T.F., Sandra Kooij J.J. (2015). Circadian rhythm disruption as a link between Attention-Deficit/Hyperactivity Disorder and obesity?. J. Psychosom. Res..

[86] Khalife N., Kantomaa M., Glover V., Tammelin T., Laitinen J., Ebeling H., Hurtig T., Jarvelin M.-R., Rodriguez A. (2014). Childhood Attention-Deficit/Hyperactivity Disorder Symptoms Are Risk Factors for Obesity and Physical Inactivity in Adolescence. J. Am. Acad. Child Adolesc. Psychiatry.

[87] Cook B.G., Li D., Heinrich K.M. (2015). Obesity, Physical Activity, and Sedentary Behavior of Youth With Learning Disabilities and ADHD. J. Learn. Disabil..

[88] Cortese S., Castellanos F.X. (2014). The relationship between ADHD and obesity: Implications for therapy. Expert Rev. Neurother..

[89] Levy L.D., Fleming J.P., Klar D. (2009). Treatment of refractory obesity in severely obese adults following management of newly diagnosed attention deficit hyperactivity disorder. Int. J. Obes..

[90] Verbeken S., Braet C., Goossens L., van der Oord S. (2013). Executive function training with game elements for obese children: A novel treatment to enhance self-regulatory abilities for weight-control. Behav. Res. Ther..

[91] Akiskal K.K., Akiskal H.S. (2005). The theoretical underpinnings of affective temperaments: Implications for evolutionary foundations of bipolar disorder and human nature. J. Affect. Disord..

[92] Rovai L., Maremmani A.G.I., Rugani F., Bacciardi S., Pacini M., Dell’osso L., Akiskal H.S., Maremmani I. (2013). Do Akiskal & Mallya’s affective temperaments belong to the domain of pathology or to that of normality?. Eur. Rev. Med. Pharmacol. Sci..

[93] Cloninger C.R., Svrakic D.M., Przybeck T.R. (1993). A psychobiological model of temperament and character. Arch. Gen. Psychiatry.

[94] Perugi G., Maremmani I., Toni C., Madaro D., Mata B., Akiskal H.S. (2001). The contrasting influence of depressive and hyperthymic temperaments on psychometrically derived manic subtypes. Psychiatry Res..

[95] Akiskal H.S., Hantouche E.G., Allilaire J.F. (2003). Bipolar II with and without cyclothymic temperament: “dark” and “sunny” expressions of soft bipolarity. J. Affect. Disord..

[96] Boulanger H., Tebeka S., Girod C., Lloret-Linares C., Meheust J., Scott J., Guillaume S., Courtet P., Bellivier F., Delavest M. (2018). Binge eating behaviours in bipolar disorders. J. Affect. Disord..

[97] Alciati A., D’Ambrosio A., Foschi D., Corsi F., Mellado C., Angst J. (2007). Bipolar spectrum disorders in severely obese patients seeking surgical treatment. J. Affect. Disord..

[98] Amianto F., Lavagnino L., Leombruni P., Gastaldi F., Daga G.A., Fassino S. (2011). Hypomania across the binge eating spectrum. A study on hypomanic symptoms in full criteria and sub-threshold binge eating subjects. J. Affect. Disord..

[99] Signoretta S., Maremmani I., Liguori A., Perugi G., Akiskal H.S. (2005). Affective temperament traits measured by TEMPS-I and emotional-behavioral problems in clinically-well children, adolescents, and young adults. J. Affect. Disord..

[100] Perugi G., Toni C., Passino M.C.S., Akiskal K.K., Kaprinis S., Akiskal H.S. (2006). Bulimia nervosa in atypical depression: The mediating role of cyclothymic temperament. J. Affect. Disord..

[101] Amann B., Mergl R., Torrent C., Perugi G., Padberg F., El-Gjamal N., Laakmann G. (2009). Abnormal temperament in patients with morbid obesity seeking surgical treatment. J. Affect. Disord..

[102] Lesiewska N., Borkowska A., Junik R., Kamińska A., Pulkowska-Ulfig J., Tretyn A., Bieliński M. (2019). The Association Between Affective Temperament Traits and Dopamine Genes in Obese Population. Int. J. Mol. Sci..

[103] Ramacciotti C.E., Paoli R.A., Ciapparelli A., Marcacci G., Placidi G.E., Dell’Osso L., Garfinkel P.E. (2004). Affective temperament in the eating disorders. Eat. Weight Disord..

[104] Marzola E., Fassino S., Amianto F., Abbate-Daga G. (2017). Affective temperaments in anorexia nervosa: The relevance of depressive and anxious traits. J. Affect. Disord..

[105] Feki I., Sellami R., Chouayakh S. (2016). Eating Disorders and Cyclothymic Temperament: A Cross-Sectional Study on Tunisian Students. Bipolar Disord. Open Access.

[106] Friedman N.P., Miyake A. (2017). Unity and diversity of executive functions: Individual differences as a window on cognitive structure. Cortex.

[107] Favieri F., Forte G., Casagrande M. (2019). The executive functions in overweight and obesity: A systematic review of neuropsychological cross-sectional and longitudinal studies. Front. Psychol..

[108] Raman J., Hay P., Tchanturia K., Smith E. (2018). A randomised controlled trial of manualized cognitive remediation therapy in adult obesity. Appetite.

[109] Allom V., Mullan B., Smith E., Hay P., Raman J. (2018). Breaking bad habits by improving executive function in individuals with obesity. BMC Public Health.

[110] Spitznagel M.B., Garcia S., Miller L.A., Strain G., Devlin M., Wing R., Cohen R., Paul R., Crosby R., Mitchell J.E. (2013). Cognitive function predicts weight loss after bariatric surgery. Surg. Obes. Relat. Dis..

[111] Dassen F.C.M., Houben K., Allom V., Jansen A. (2018). Self-regulation and obesity: The role of executive function and delay discounting in the prediction of weight loss. J. Behav. Med..

[112] Reinelt T., Petermann F., Bauer F., Bauer C.P. (2020). Emotion regulation strategies predict weight loss during an inpatient obesity treatment for adolescents. Obes. Sci. Pract..

[113] van Egmond-Fröhlich A.W.A., Weghuber D., de Zwaan M. (2012). Association of Symptoms of Attention-Deficit/Hyperactivity Disorder with Physical Activity, Media Time, and Food Intake in Children and Adolescents. PLoS ONE.

[114] Veronese N., Facchini S., Stubbs B., Luchini C., Solmi M., Manzato E., Sergi G., Maggi S., Cosco T., Fontana L. (2017). Weight loss is associated with improvements in cognitive function among overweight and obese people: A systematic review and meta-analysis. Neurosci. Biobehav. Rev..

[115] Gunstad J., Strain G., Devlin M.J., Wing R., Cohen R.A., Paul R.H., Crosby R.D., Mitchell J.E. (2011). Improved memory function 12 weeks after bariatric surgery. Surg. Obes. Relat. Dis..

[116] Vantieghem S., Bautmans I., De Guchtenaere A., Tanghe A., Provyn S. (2018). Improved cognitive functioning in obese adolescents after a 30-week inpatient weight loss program. Pediatr. Res..

[117] Moeller F.G., Barratt E.S., Dougherty D.M., Schmitz J.M., Swann A.C. (2001). Psychiatric Aspects of Impulsivity. Am. J. Psychiatry.

[118] Nasser J.A., Gluck M.E., Geliebter A. (2004). Impulsivity and test meal intake in obese binge eating women. Appetite.

[119] Jauregi A., Kessler K., Hassel S. (2018). Linking cognitive measures of response inhibition and reward sensitivity to trait impulsivity. Front. Psychol..

[120] Bénard M., Camilleri G.M., Etilé F., Méjean C., Bellisle F., Reach G., Hercberg S., Péneau S. (2017). Association between Impulsivity and Weight Status in a General Population. Nutrients.

[121] Giel K.E., Teufel M., Junne F., Zipfel S., Schag K. (2017). Food-Related Impulsivity in Obesity and Binge Eating Disorder-A Systematic Update of the Evidence. Nutrients.

[122] Meule A. (2013). Impulsivity and overeating: A closer look at the subscales of the Barratt Impulsiveness Scale. Front. Psychol..

[123] Liu Y., Zhao J., Zhang X., Gao X., Xu W., Chen H. (2019). Overweight adults are more impulsive than normal weight adults: Evidence from ERPs during a chocolate-related delayed discounting task. Neuropsychologia.

[124] Oliva R., Morys F., Horstmann A., Castiello U., Begliomini C. (2019). The impulsive brain: Neural underpinnings of binge eating behavior in normal-weight adults. Appetite.

[125] Fernandes J., Ferreira-Santos F., Miller K., Torres S. (2018). Emotional processing in obesity: A systematic review and exploratory meta-analysis. Obes. Rev..

[126] Leehr E.J., Krohmer K., Schag K., Dresler T., Zipfel S., Giel K.E. (2015). Emotion regulation model in binge eating disorder and obesity—A systematic review. Neurosci. Biobehav. Rev..

[127] Kittel R., Brauhardt A., Hilbert A. (2015). Cognitive and emotional functioning in binge-eating disorder: A systematic review. Int. J. Eat. Disord..

[128] Burton A.L., Abbott M.J. (2019). Processes and pathways to binge eating: Development of an integrated cognitive and behavioural model of binge eating. J. Eat. Disord..

[129] McElroy S.L., Crow S., Blom T.J., Cuellar-Barboza A.B., Prieto M.L., Veldic M., Winham S.J., Bobo W.V., Geske J., Seymour L.R. (2016). Clinical features of bipolar spectrum with binge eating behaviour. J. Affect. Disord..

[130] Gearhardt A.N., Corbin W.R., Brownell K.D. (2009). Preliminary validation of the Yale Food Addiction Scale. Appetite.

[131] Burrows T., Skinner J., McKenna R., Rollo M. (2017). Food Addiction, Binge Eating Disorder, and Obesity: Is There a Relationship?. Behav. Sci..

[132] Gordon E.L., Ariel-Donges A.H., Bauman V., Merlo L.J. (2018). What Is the Evidence for “Food Addiction?” A Systematic Review. Nutrients.

[133] Ziauddeen H., Fletcher P.C. (2013). Is food addiction a valid and useful concept?. Obes. Rev..

[134] Pedram P., Wadden D., Amini P., Gulliver W., Randell E., Cahill F., Vasdev S., Goodridge A., Carter J., Zhai G. (2013). Food Addiction: Its Prevalence and Significant Association with Obesity in the General Population. PLoS ONE.

[135] Meule A., Muller A., Gearhardt A.N., Blechert J. (2017). German version of the Yale Food Addiction Scale 2.0: Prevalence and correlates of “food addiction” in students and obese individuals. Appetite.

[136] Pursey K.M., Stanwell P., Gearhardt A.N., Collins C.E., Burrows T.L. (2014). The prevalence of food addiction as assessed by the Yale Food Addiction Scale: A systematic review. Nutrients.

[137] Imperatori C., Innamorati M., Contardi A., Continisio M., Tamburello S., Lamis D.A., Tamburello A., Fabbricatore M. (2014). The association among food addiction, binge eating severity and psychopathology in obese and overweight patients attending low-energy-diet therapy. Compr. Psychiatry.

[138] Muller A., Leukefeld C., Hase C., Gruner-Labitzke K., Mall J.W., Kohler H., de Zwaan M. (2018). Food addiction and other addictive behaviours in bariatric surgery candidates. Eur. Eat. Disord. Rev..

[139] Hauck C., Weiß A., Schulte E.M., Meule A., Ellrott T. (2017). Prevalence of “Food Addiction’’ as Measured with the Yale Food Addiction Scale 2.0 in a Representative German Sample and Its Association with Sex, Age and Weight Categories. Obes. Facts.

[140] Imperatori C., Fabbricatore M., Vumbaca V., Innamorati M., Contardi A., Farina B. (2016). Food Addiction: Definition, measurement and prevalence in healthy subjects and in patients with eating disorders. Riv. Psichiatr..

[141] Luigjes J., Lorenzetti V., de Haan S., Youssef G.J., Murawski C., Sjoerds Z., van den Brink W., Denys D., Fontenelle L.F., Yücel M. (2019). Defining Compulsive Behavior. Neuropsychol. Rev..

[142] Gearhardt A.N., White M.A., Masheb R.M., Morgan P.T., Crosby R.D., Grilo C.M. (2012). An examination of the food addiction construct in obese patients with binge eating disorder. Int. J. Eat. Disord..

[143] Backman O., Stockeld D., Rasmussen F., Naslund E., Marsk R. (2016). Alcohol and substance abuse, depression and suicide attempts after Roux-en-Y gastric bypass surgery. Br. J. Surg..

[144] Mitchell J.E., Steffen K., Engel S., King W.C., Chen J.-Y., Winters K., Sogg S., Sondag C., Kalarchian M., Elder K. (2015). Addictive disorders after Roux-en-Y gastric bypass. Surg. Obes. Relat. Dis..

